# Implications of Pericardial, Visceral and Subcutaneous Adipose Tissue on Vascular Inflammation Measured Using ^18^FDG-PET/CT

**DOI:** 10.1371/journal.pone.0135294

**Published:** 2015-08-13

**Authors:** Ho Cheol Hong, Soon Young Hwang, Soyeon Park, Ja Young Ryu, Hae Yoon Choi, Hye Jin Yoo, Ji-A Seo, Sin Gon Kim, Nan Hee Kim, Sei Hyun Baik, Dong Seop Choi, Sungeun Kim, Kyung Mook Choi

**Affiliations:** 1 Division of Endocrinology and Metabolism, Department of Internal Medicine, College of Medicine, Korea University, Seoul, Korea; 2 Department of Biostatistics, College of Medicine, Korea University, Seoul, Korea; 3 Department of Nuclear Medicine, College of Medicine, Korea University, Seoul, Korea; IRCCS Scientific Institute and Regional General Hospital Casa Sollievo della Sofferenza Opera di Padre Pio da Pietrelcina, ITALY

## Abstract

**Objective:**

Pericardial adipose tissue (PAT) is associated with adverse cardiometabolic risk factors and cardiovascular disease (CVD). However, the relative implications of PAT, abdominal visceral and subcutaneous adipose tissue on vascular inflammation have not been explored.

**Method and Results:**

We compared the association of PAT, abdominal visceral fat area (VFA), and subcutaneous fat area (SFA) with vascular inflammation, represented as the target-to-background ratio (TBR), the blood-normalized standardized uptake value measured using ^18^F-Fluorodeoxyglucose Positron Emission Tomography (^18^FDG-PET) in 93 men and women without diabetes or CVD. Age- and sex-adjusted correlation analysis showed that PAT, VFA, and SFA were positively associated with most cardiometabolic risk factors, including systolic blood pressure, LDL-cholesterol, triglycerides, glucose, insulin resistance and high sensitive C-reactive proteins (hsCRP), whereas they were negatively associated with HDL-cholesterol. In particular, the maximum TBR (maxTBR) values were positively correlated with PAT and VFA (*r* = 0.48 and *r* = 0.45, respectively; both *P* <0.001), whereas SFA showed a relatively weak positive relationship with maxTBR level (*r* = 0.31, *P* = 0.003).

**Conclusion:**

This study demonstrated that both PAT and VFA are significantly and similarly associated with vascular inflammation and various cardiometabolic risk profiles.

## Introduction

Obesity is closely related to glucose and lipid metabolism, and is a well-established risk factor for atherosclerosis and cardiovascular disease (CVD) [1]. Adipose depots in different locations may confer different metabolic risk and cardio-metabolic burdens for the development of CVD. For example, abdominal visceral adipose tissue (VAT) is more closely associated with metabolic syndrome and cardiovascular mortality than subcutaneous adipose tissue (SAT) [2]. Even more, several studies suggest that SAT may serve as a buffer that protects other tissues from ectopic lipotoxic effects [3].

The heart has its own visceral fat depots between the myocardium and the visceral pericardium [epicardial adipose tissue (EAT)] and around the paracardial area (paracardial adipose tissue) [4]. Recently, pericardial adipose tissue (PAT) [EAT plus paracardial adipose tissue] has been recognized as a novel risk factor for coronary artery disease (CAD). In the Framingham Heart Study, Rosito et al. [5] found that pericardial fat was associated with coronary artery calcification (CAC) after multivariable analysis and visceral fat area (VFA) adjustment. In the Multi-ethnic Study of Atherosclerosis (MESA), PAT was associated with increased carotid stiffness, independent of traditional CVD risk factors and obesity [6]. Miao et al. [7] reported that PAT correlated significantly with the degree of coronary artery eccentricity an index of plaque burden, as measured using magnetic resonance (MR) imaging in 183 participants from the MESA study. Furthermore, Greif et al. [8] also reported that increased PAT is associated with coronary atherosclerosis, hypoadiponectinemia, and systemic inflammatory markers. Patients who had only non-calcified plaques had significantly increased PAT, indicating that PAT quantification may be useful to identify patients with CAD, even in the absence of coronary calcification.

Chronic low-grade inflammation is associated with chronic metabolic disorders, and atherosclerosis is now recognized as an inflammatory disorder [9]. Vascular culprit lesions for acute coronary syndrome or myocardial infarction are associated with a high degree of plaque inflammation [10]. Positron Emission Tomography with ^18^F-Fluorodeoxyglucose (^18^FDG-PET) is a novel imaging technique to identify vascular inflammation, and indicates macrophage infiltration in vasculature [11]. In our previous studies, ^18^FDG-PET revealed that patients with impaired glucose metabolism or elevated high sensitive C-reactive protein (hsCRP) had significantly higher maximum target-to-background ratio (TBR) values, which indicates vascular inflammation, than their control groups [12, 13]. In contrast, TBR values showed a negative relationship with serum adiponectin, which is an anti-inflammatory and anti-atherogenic adipokine [14].

PAT/EAT affect the cardiovascular system by secreting pro- and anti-inflammatory adipokines in paracrine or vasocrine manners [4]. The role of PAT in the development of CAD may be due to its close anatomical proximity to the myocardium and vasculature [15]. PAT/EAT may have different metabolic features from other visceral fat depots, especially with respect to adipocyte size and free fatty acid metabolism [16]. However, no previous studies have explored the association of PAT or VFA with vascular inflammation, which may reflect plaque vulnerability. Moreover, few studies investigate the importance of PAT as a cardiovascular risk factor compared to other adipose tissue depots, such as the abdominal VFA or the subcutaneous fat area (SFA).

In the present study, we compared vascular inflammation measured by ^18^FDG-PET/CT between PAT, VFA, and SFA and investigated their relationships with conventional and novel cardiometabolic risk parameters in individuals without a history of CAD or diabetes.

## Materials and Methods

### Study design and participants

We recruited patients who were self-referred for a routine health check-up at the Health Promotion Center of Korea, University Guro Hospital, between June 2008 and March 2009. Subjects were excluded from this study if they met any of the following criteria: history of CVD (myocardial infarction, unstable angina, stroke, or cardiovascular revascularization); diabetes; stage 2 hypertension (resting blood pressure ≥160/100 mmHg); any lipid-lowering therapies or postmenopausal hormone replacement therapy during the 6-month period before enrollment; history of inflammatory conditions such as aortitis and vasculitis; any medications that might affect inflammatory status, including steroid and non-steroidal anti-inflammatory drug within 6 months of the study; malignant disease; or severe renal or hepatic disease. After excluding ineligible subjects, we analyzed on 93 participants (53 men and 40 women). All participants provided written informed consent, and the Korea University Institutional Review Board approved this study protocol, in accordance with the Declaration of Helsinki of the World Medical Association.

### Anthropometric and laboratory measurements

Body mass index (BMI) was calculated as the weight/height^2^ (kg/m^2^), and waist circumference was measured at the midpoint between the lower border of the rib cage and the iliac crest. All blood samples were obtained in the morning after a 12-hour overnight fast and were immediately stored at –80°C. Serum triglycerides and high-density lipoprotein cholesterol (HDL-C) were measured enzymatically using a chemistry analyzer (Hitachi 747; Hitachi Inc.). The low-density lipoprotein cholesterol (LDL-C) concentration was estimated using the Friedewald formula [17]. A glucose oxidase method was used to measure plasma glucose. hsCRP levels were measured by Latex-enhanced Turbidimetric Immunoassay (HiSens hsCRP LTIA; HBI Co., Ltd.), with an interassay coefficient of variation of 7.2%.

### ^18^F-FDG PET/CT

PET/CT was performed using the Gemini TF 16-slice PET/CT scanner (Philips). The TF scanner is a new high-performance, time-of-flight–capable, fully 3-dimensional PET scanner using lutetium yttrium oxyorthosilicate crystals [18]. After the patients had fasted for at least 12-hours, ^18^F-FDG (5.19 MBq/kg) was injected intravenously, and patients rested in a quiet room for 60 minutes. Whole-body PET images (from below the cerebellum to the inguinal region) were acquired for 10 minutes (1 minute per bed position) and the resulting images were analyzed on a dedicated workstation (Extended Brilliance Workspace 3.5 with PET/CT viewer for automated image registration; Philips). The right carotid ^18^F-FDG uptake was measured along the length of the right carotid vessel, starting at the bifurcation and extending inferiorly and superiorly every 4 mm, for a total 10 consecutive PET/CT images for each subject. Arterial ^18^F-FDG uptake was quantified by a region of interest (ROI) around each artery on every slice of the coregistered transaxial PET/CT images. The ROI was fitted to the artery wall on each axial slice, and coronal and sagittal views were used to ensure that the ^18^F-FDG uptake was from the artery. The standardized uptake value (SUV) is the decay-corrected tissue concentration of ^18^F-FDG (in kBq/mL) divided by the injected dose per body weight (kBq/g). On each image slice, the mean and maximum SUVs of the ROI were measured as the mean and maximum pixel activity. The SUVs for all 10 slices within the right carotid artery were averaged to calculate the mean and maximum SUVs for each participant. Next, the arterial SUV was divided by the blood-pool SUV measured from the jugular vein for normalization; thereby, a mean and maximum TBR value was acquired for each subject by an experienced radiologist in the Department of Nuclear Medicine (S.K.) [19].

### Measurement of PAT, VFA, and SFA

The VFA, SFA and PAT volumes were measured on non-contrast enhanced CT scans using volume analysis software of the workstation (Extended Brilliance Workspace 3.5, with PET/CT viewer; Philips). The vertical PAT ranged from the bifurcation of the pulmonary artery superiorly, to the cardiac apex over the diaphragm inferiorly. The anterior border was defined by the posterior aspect of sternum and the posterior border was defined by the esophagus and the descending aorta. The ROI containing the heart and the surrounding adipose tissue was assessed by manually tracing axial slices. PAT images ranged from –190 to –30 Hounsfield units (HU) to isolate the adipose tissue containing voxels. The adipose tissue voxels were then summed to obtain PAT volume. The VFA and total abdominal fat area were calculated using the CT slice scan image at the umbilicus level. VFA was quantified by manual tracing of the intra-abdominal cavity from the internal aspect of the abdominal and oblique muscle walls and the posterior aspect of the vertebral body. The subcutaneous fat area was calculated by subtracting the VFA from the total abdominal fat area. A threshold of –190 to –30 HU was also applied to isolate fat-containing voxels.

### Statistical analyses

Data are expressed as mean ± standard deviation, median and interquartile range (25%-75%), or percentage. Differences between groups were tested using the Student’s t-test or the Mann-Whitney U-test, and the χ^2^-test was used to test for differences in the distribution of categorical variables. Each variable was examined for normal distribution by using the Shapiro-Wilk normality test. An age and sex-adjusted Spearman partial correlation analysis of VFA, SFA and PAT with other metabolic variables was conducted. We further divided the study subjects into 3 groups according to the tertile of maximum TBR (maxTBR). We then compared the cardio-metabolic risk parameters of these 3 groups to estimate the significance of any statistical associations between tertiles of maxTBR by using the analysis of variance (ANOVA) or the Kruskal-Wallis test and the χ^2^-test as appropriate. For multiple comparisons, we used the Tukey-Kramer test for variables with normal distribution and the Mann-Whitney test for those without normal distribution, respectively. Also, we adjusted the *P*-values of the Mann-Whitney U-test by using the Bonferoni correction in multiple comparisons. After adjusting for age and sex, we compared the cardio-metabolic risk parameters by using the analysis of covariance (ANCOVA) for variables with normal distribution. For non-normal continuous variables, ANCOVA was conducted after log-transformation. And, a logistic regression analysis was used for categorical variables. A *P*-value <0.05 was considered statistically significant in all analyses. All statistical results were based on two-sided tests. Data were analyzed using SAS for Windows (version 9.20, SAS Institute Inc., Cary, NC, USA).

## Results

### Characteristics of study participants

The characteristics of all study subjects are presented in Table 1. The mean age of men and women was 48.8 ± 8.9 years and 53.2 ± 9.9 years, respectively. PAT was slightly larger in men than in women (238.4 [171.2, 287.0] cm^3^ vs. 186.0 [144.4, 267.4] cm^3^, *P* = 0.022). In addition, VFA was larger in men than in women (113.9 ± 39.1 cm^2^ vs. 93.3 ± 33.4 cm^2^, *P* = 0.009), whereas SFA was greater in women than in men (157.7 ± 49.4 cm^2^ vs. 205.5 ± 61.3 cm^2^, *P* <0.001). On the other hand, hsCRP and HOMA-IR of study subjects were not significantly different between men and women.

**Table 1:** Clinical Characteristics of Study Participants.

	Male (n = 53)	Female (n = 40)	*P*
Age (years)	48.8 ± 8.9	53.2 ± 9.9	0.026
BMI (kg/m^2^)	24.8 ± 2.9	23.3 ± 3.0	0.017
Waist circumference (cm)	87.4 ± 6.3	79.2 ± 7.6	<0.001
SBP (mmHg)	125.0 (118.5, 131.0)	122.0 (113.5, 136.5)	0.456
DBP (mmHg)	85.1 ± 10.4	80.2 ± 11.5	0.036
Triglyceride (mmol/L)	1.4 (0.9, 2.2)	0.9 (0.6, 1.6)	0.006
HDL-C (mmol/L)	1.2 (1.1, 1.5)	1.2 (1.0, 1.4)	0.576
LDL-C (mmol/L)	3.5 ± 0.9	3.1 ± 1.2	0.103
Glucose (mmol/L)	5.3 ± 0.7	4.6 ± 1.0	0.001
hsCRP (mg/L)	2.2 (0.5, 3.5)	0.8 (0.4, 2.9)	0.141
HOMA-IR	0.7 (0.3, 1.2)	0.5 (0.3, 0.9)	0.167
Current smoker	24 (45.3)	3 (7.5)	<0.001
Maximum TBR	1.3 (1.2, 1.5)	1.2 (1.1, 1.4)	0.037
VFA (cm^2^)	113.9 ± 39.1	93.3 ± 33.4	0.009
SFA (cm^2^)	157.7 ± 49.4	205.5 ± 61.3	<0.001
PAT (cm^3^)	238.4 (171.2, 287.0)	186.0 (144.4, 267.4)	0.022

BMI, body mass index; SBP, systolic blood pressure; DBP, diastolic blood pressure; HDL-C, high-density lipoprotein cholesterol; LDL-C, low-density lipoprotein cholesterol; hsCRP, high sensitivity C-reactive protein; HOMA-IR, homeostasis model assessment insulin resistance; TBR, target-to-background ratio; VFA, (abdominal) visceral fat area; SFA, (abdominal) subcutaneous fat area; PAT, pericardial adipose tissue.

Data with a normal distribution are shown as “mean ± SD”, and those without a normal distribution are shown as “median (interquartile range)”.

Only current smoker is expressed in N (%).

### Correlation of VFA, SFA and PAT with cardiometabolic risk factors

**Fig 1:**
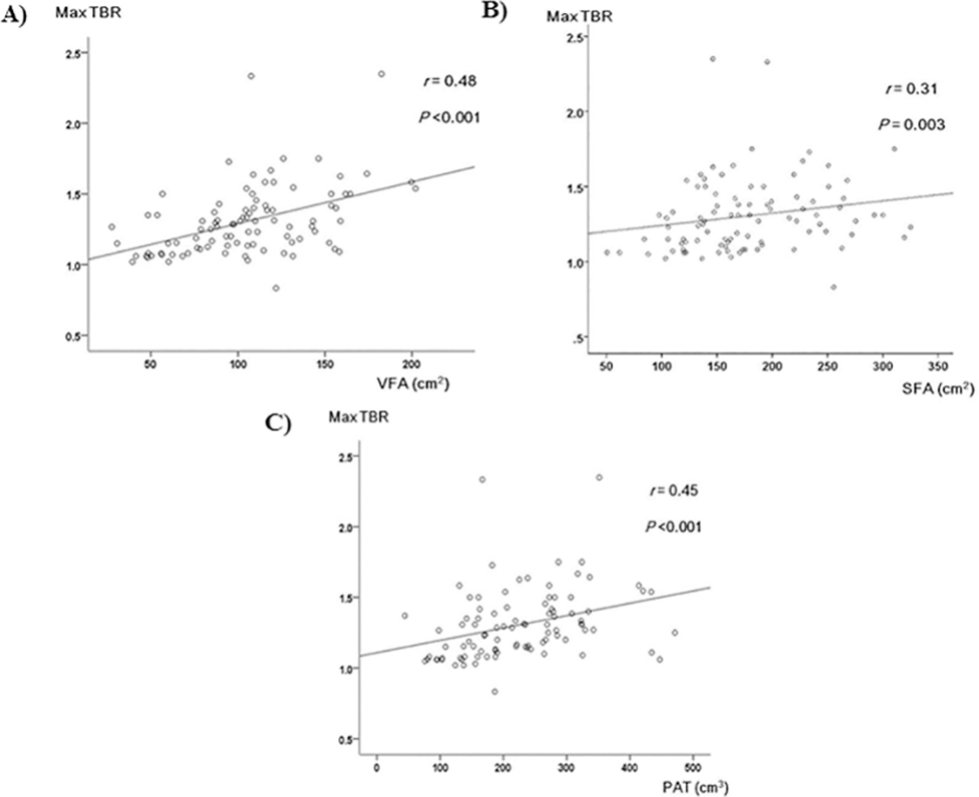
Scatterplot of maximum TBR values with A) abdominal visceral fat area (VFA), B) abdominal subcutaneous fat area (SFA), and C) pericardial adipose tissue volume (PAT).

Table 2 shows the correlation analysis of VFA, SFA and PAT with other major metabolic variables. After adjusting for age and sex, VFA, SFA, and PAT were positively correlated with most cardiometabolic risk factors, including obesity parameters, hypertension, dyslipidemia, hyperglycemia, HOMA-IR, and hsCRP. On the other hand, HDL-C was negatively correlated with all adipose depots. PAT showed a significant relationship with both VFA (*r* = 0.69, *P* <0.001) and SFA (*r* = 0.56, *P* <0.001). In particular, the maxTBR value was strongly and positively correlated with VFA and PAT (*r* = 0.48 and 0.45, respectively; both *P* <0.001), but SFA showed a relatively weak relationship with maxTBR levels (*r* = 0.31, *P* = 0.003) (Table 2, Fig 1), despite its close relationships with VFA (*r* = 0.52, *P* <0.001) and PAT.

**Table 2:** Spearman Partial Correlation Analysis of Abdominal Visceral Fat Area (VFA), Abdominal Subcutaneous Fat Area (SFA), and Pericardial Adipose Tissue Volume (PAT) With Cardiometabolic Parameters, after Adjusting for Age and Sex.

	VFA		SFA		PAT	
	*r*	*P*	*r*	*P*	*r*	*P*
BMI	0.58	<0.001	0.74	<0.001	0.60	<0.001
Waist circumference	0.67	<0.001	0.67	<0.001	0.62	<0.001
SBP	0.25	0.017	0.22	0.038	0.28	0.008
DBP	0.31	0.003	0.16	0.124	0.18	0.091
LDL-C	0.37	<0.001	0.34	0.001	0.29	0.006
HDL-C	−0.35	0.001	−0.25	0.019	−0.35	0.001
Triglyceride	0.45	<0.001	0.38	<0.001	0.52	<0.001
Glucose	0.39	<0.001	0.35	0.001	0.31	0.003
hsCRP	0.45	<0.001	0.33	0.001	0.35	0.001
HOMA-IR	0.53	<0.001	0.50	<0.001	0.41	<0.001

BMI, body mass index; SBP, systolic blood pressure; DBP, diastolic blood pressure; HDL-C, high-density lipoprotein cholesterol; LDL-C, low-density lipoprotein cholesterol; hsCRP, high sensitivity C-reactive protein; HOMA-IR, homeostasis model assessment insulin resistance; TBR, target-to-background ratio.

### Cardiovascular risk factors stratified by tertile of maximum TBR value

BMI, waist circumference, SBP, DBP, triglyceride, LDL-C, hsCRP, HOMA-IR and maxTBR values were increased with maxTBR tertiles. And, these trends did not change even after adjusting for age and sex (Table 3). Interestingly, VFA, SFA and PAT showed a stepwise increase with increasing maxTBR tertiles (*P* <0.001, *P* = 0.001 and *P* <0.001, respectively).

**Table 3:** Anthropometric, Metabolic and Cardiovascular Risk Parameters of Study Subjects by Tertiles of Maximum Target-to-Background Ratio (maxTBR) value.

	MaxTBR T1 (0.83–1.15)	MaxTBR T2 (1.16–1.35)	MaxTBR T3 (1.36–2.35)	*P*	*P’*
Sex, male (%)	16 (50.0)	16 (53.3)	21 (67.7)	0.322	
Age (years)	50.8 ± 8.6	49.0 ± 10.0	52.1 ± 10.2	0.461	
BMI (kg/m^2^)	22.3 (19.2, 24.2)^a^	25.0 (23.7, 26.8)^b^	25.4 (23.7, 26.9)^b^	<0.001	<0.001
Waist circumference (cm)	79.1 ± 8.0^a^	85.2 ± 7.0^b^	87.5 ± 6.5^b^	<0.001	<0.001
SBP (mmHg)	120.0 (111.0, 125.0)^a^	126.0 (118.0, 133.0)^ab^	127.0 (118.0, 143.0)^b^	0.018	0.005
DBP (mmHg)	78.4 ± 8.8^a^	82.6 ± 10.5^ab^	87.8 ± 11.9^b^	0.003	0.009
Triglycerides (mmol/L)	0.8 (0.6, 1.0)^a^	1.6 (1.0, 2.3)^b^	1.4 (1.2, 2.2)^b^	<0.001	<0.001
HDL-C (mmol/L)	1.3 ± 0.4	1.2 ± 0.3	1.2 ± 0.3	0.516	0.489
LDL-C (mmol/L)	2.6 (2.0, 3.8)^a^	2.9 (2.4, 4.3)^ab^	3.8 (3.4, 4.3)^b^	<0.001	<0.001
Glucose (mmol/L)	4.5 ± 0.9^a^	5.0 ± 0.8^b^	5.5 ± 0.7^b^	<0.001	<0.001
hsCRP (mg/L)	0.4 (0.2, 0.8)^a^	1.5 (0.5, 3.1)^b^	3.2 (2.6, 4.8)^c^	<0.001	<0.001
HOMA-IR	0.3 (0.2, 0.7)^a^	0.8 (0.5, 1.2)^b^	1.0 (0.5, 1.6)^b^	<0.001	<0.001
Current smoker (%)	10 (31.2)	8 (26.7)	9 (29.0)	0.924	0.688
VFA (cm^2^)	84.8 ± 35.9^a^	101.7 ± 30.0^a^	129.2 ± 34.2^b^	<0.001	<0.001
SFA (cm^2^)	146.4 ± 48.4^a^	197.0 ± 67.2^b^	193.0 ± 49.0^b^	0.001	<0.001
PAT (cm^3^)	157.4 (116.3, 205.1)^a^	233.6 (171.2, 285.1)^b^	272.6 (185.5, 317.5)^b^	<0.001	<0.001

BMI, body mass index; SBP, systolic blood pressure; DBP, diastolic blood pressure; HDL-C, high-density lipoprotein cholesterol; LDL-C, low-density lipoprotein cholesterol; hsCRP, high sensitivity C-reactive protein; HOMA-IR, homeostasis model assessment insulin resistance; VFA, (abdominal) visceral fat area; SFA, (abdominal) subcutaneous fat area; PAT, pericardial adipose tissue.

Data are mean ± SD, median (interquartile range), or N (%).

Means or medians with the different letter are significantly different.

The variables expressed as medians (interquartile range) were assessed by the the Mann-Withney test in multiple comparisons, and the *P*-values were adjusted by the Bonferoni correction (P <0.017 was considered statistically significant).

*P*’ values were calculated after adjusting for age and sex by using the analysis of covariance (ANCOVA) for continuous variables and a logistic regression analysis for categorical variables.

## Discussion

The terminology distinguishing different fat depots around heart is somewhat confusing [3]. In general, EAT has been defined as the intra-epicardial fat depot between the myocardium and the visceral pericardium. PAT, also known as extra-pericardial adipose tissue, has been defined to include both EAT and the fat depot outside the visceral pericardium [20]. Post mortem studies have shown that EAT covers 80% of the heart’s surface and constitutes up to 20% of the total ventricular weight of the human heart [21]. Greif et al. [8] reported an excellent correlation between EAT and PAT (*r* = 0.97), although PAT had better reproducibility (interobserver variability 8%) than EAT (15%). PAT/EAT can generate various bioactive molecules that may affect cardiovascular health [3]. Previous studies have shown the association between PAT/EAT and early-stage atherosclerosis and plaque formation. The accumulation of pericardial fat is associated with impairment in left ventricular function, independent of other factors such as hypertension or diabetes [22]. In a study using multi-detector computed tomography (MDCT), increased pericardial fat volume was an independent risk factor for coronary artery stenosis, even after adjusting for confounding factors [23]. Moreover, Schlett et al. [24] found that patients with high-risk coronary lesions have nearly twice the PAT as those without CAD, independent of clinical characteristics and general obesity. In a nested case-control study including asymptomatic patients without CAD, individuals experiencing major adverse cardiovascular events (MACE) had a significantly higher pericardial fat burden compared with event-free control subjects even with similar cardiovascular risk profiles [25]. Based on their 5.4-year cohort study in 145 patients with stable CAD, Greif et al. [15] suggested that PAT measurement increases the predictive power of CAC for the development of cardiovascular events. In the present study, we first showed the significant implications of PAT and VFA with FDG uptake values measured using ^18^FDG-PET/CT, which indicates macrophage infiltration within carotid vasculature.

Several pathogenic mechanisms that may mediate the relationship between PAT and atherosclerosis have been suggested. In individuals without CAD or diabetes, PAT was associated with insulin resistance, independent of BMI and waist circumference [26]. This is in agreement with our observation that both PAT and VFA were significantly associated with insulin resistance. Additionally, both PAT and VFA showed close relationships with several components of metabolic syndrome, such as hypertension, hyperglycemia and dyslipidemia, which is consistent with previous studies [3, 8]. Pericardial fat, which has greater lipolytic activity than subcutaneous fat, releases excessive free fatty acids to promote atherosclerosis. Furthermore, recent studies support visceral adipose tissue in obesity as an important source of inflammation, which is characterized by increased infiltration of macrophages, lymphocytes, and mast cells [27]. Pericardial fat produces more pro-inflammatory adipokines, such as tumor necrosis factor-α (TNF-α), interleukin-6 (IL-6), and monocyte chemotactic protein-1 (MCP-1), than subcutaneous fat [28]. Konishi et al. [29] examined the association between CAD and inflammation in pericardial fat measured by immunohistochemical stain in 39 autopsy cases. They found that pericardial fat inflammation was significantly associated with the presence of CAD, independent of risk factors such as hypertension, dyslipidemia, and diabetes. Vela et al. [30] have shown that macrophage infiltration in the peri-adventitial fat of rupture-prone vulnerable lipid-rich plaques is higher than in fibrocalcific plaques. In the Framingham Offspring Study, multiple markers of inflammation, such as CRP, IL-6, and oxidative stress, were correlated with PAT [31]. Mazurek et al. [32, 33] reported that inflammation of PAT increases the risk of plaque instability in the patients with acute coronary syndrome (ACS), and has a close relationship with the occurrence of atrial fibrillation. These studies indicate that inflammation of PAT is associated with cardiac electrical disturbance as well as coronary atherosclerosis. Moreover, Mazzoccoli et al. [34, 35] also reported that EAT might have a relationship with venous thromboembolism beyond arterial atherosclerosis. In this study, we confirmed the association between PAT and hsCRP, a systemic subclinical inflammatory marker. Subclinical inflammation has been established as a pivotal underlying pathogenic mechanism in the initiation, progression and rupture of atherosclerotic plaques. In contrast, adiponectin, an anti-inflammatory and anti-atherogenic adipokine, decreases with increased PAT [8]. Considering the close relationship between hsCRP, adiponectin and FDG uptake as measured by FDG-PET/CT in our previous and present results, local and systemic inflammation may link PAT with cardiovascular disorders associated with obesity [13, 14]. Lately, Alexopoulos et al. [36] reported that statin administration induced regression of EAT in hyperlipidemic post-menopausal women, and found that intensive therapy was more effective than moderate-intensity therapy. They concluded that this effect is not linked to LDL lowering and may be secondary to pleiotropic actions of statins, such as anti-inflammatory effects.

In this study, we also compared the association of vascular inflammation and traditional and novel cardiometabolic risk factors with PAT compared to other adiposity measures such as VFA and SFA. Interestingly, the magnitude of the association between PAT, vascular inflammation, and various cardiometabolic risk parameters was similar to VFA, whereas SFA had a relatively weak relationship with vascular inflammation. Considering the data showing that women have a higher SFA than men, these results might reveal an important underlying mechanism that mediates the relatively better cardiovascular outcomes in women. Our data are consistent with previous results indicating that PAT is correlated with echocardiographic measures of cardiac abnormalities, but the association is not stronger than that of other obesity indices such as waist circumference [37]. Fox et al. [38] also reported that pericardial fat is correlated with cardiovascular magnetic resonance measures, but the association was not independent or stronger than measures of visceral obesity. Moreover, Cheng et al. [39] showed that tissue levels of TNF-α, IL-6, leptin and visfatin from abdominal fat depots were significantly higher compared to those from epicardial fat in patients with CAD. Therefore, they concluded that abdominal adiposity may play a more significant role in the pathogenesis of coronary atherosclerosis than epicardial fat [39]. However, Konish et al. [40] demonstrated that pericardial fat accumulation, but not waist circumference, was significantly associated with the presence of any coronary plaques detected by coronary angiography. The exact reason for this discrepancy is not clear, our study used abdominal CT for the measurement of visceral fat, which is more accurate. Interestingly, despite the previous suggestion of protective role of SAT in cardiometabolic disorders [41, 42], the present study demonstrated that SAT had a positive association with vascular inflammation and cardiometabolic risk parameters. However, these relationships were relatively weak compared to those of PAT or VFA.

Mahabadi et al. [43] suggested that PAT is predominantly associated with coronary heart disease (CHD), whereas VFA is associated with stroke. Carotid plaque inflammation measured using ^18^FDG-PET has been reported to be related with the risk of stroke and cerebral micro-embolism [44, 45]. The present study using ^18^FDG-PET/CT examined FDG uptake that reflects vascular inflammation in carotid vasculature. Further research might be needed to explore the relative impact of PAT on regional atherosclerosis and CVD compared to other fat depots such as VAT or SAT.

Some limitations of this study need to be considered. First, the cross-sectional nature of this study did not allow us to identify causal relationships. Second, our study was comprised of Asian men and women without a history of CAD and diabetes, which limits our ability to generalize our study results to other populations with different ethnicities and characteristics.

In conclusion, the present study clearly showed that both PAT and VFA had significant associations with maxTBR values, which indicate vascular inflammation, and with other cardiometabolic risk factors as well. In addition, the magnitude of the relationship with vascular inflammation, along with the unfavorable CV risk factor profile, was similar between PAT and VFA in Korean men and women without CAD or diabetes.
